# Supplementary figures and images for: A preliminary indication that HLA-A*03:01 may be associated with visceral leishmaniasis development in people living with HIV in Ethiopia
Source: PLoS Negl Trop Dis. 2024 Sep 30;18(9):e0012000. doi: 10.1371/journal.pntd.0012000 (PMC11466428; doi:10.1371/journal.pntd.0012000)

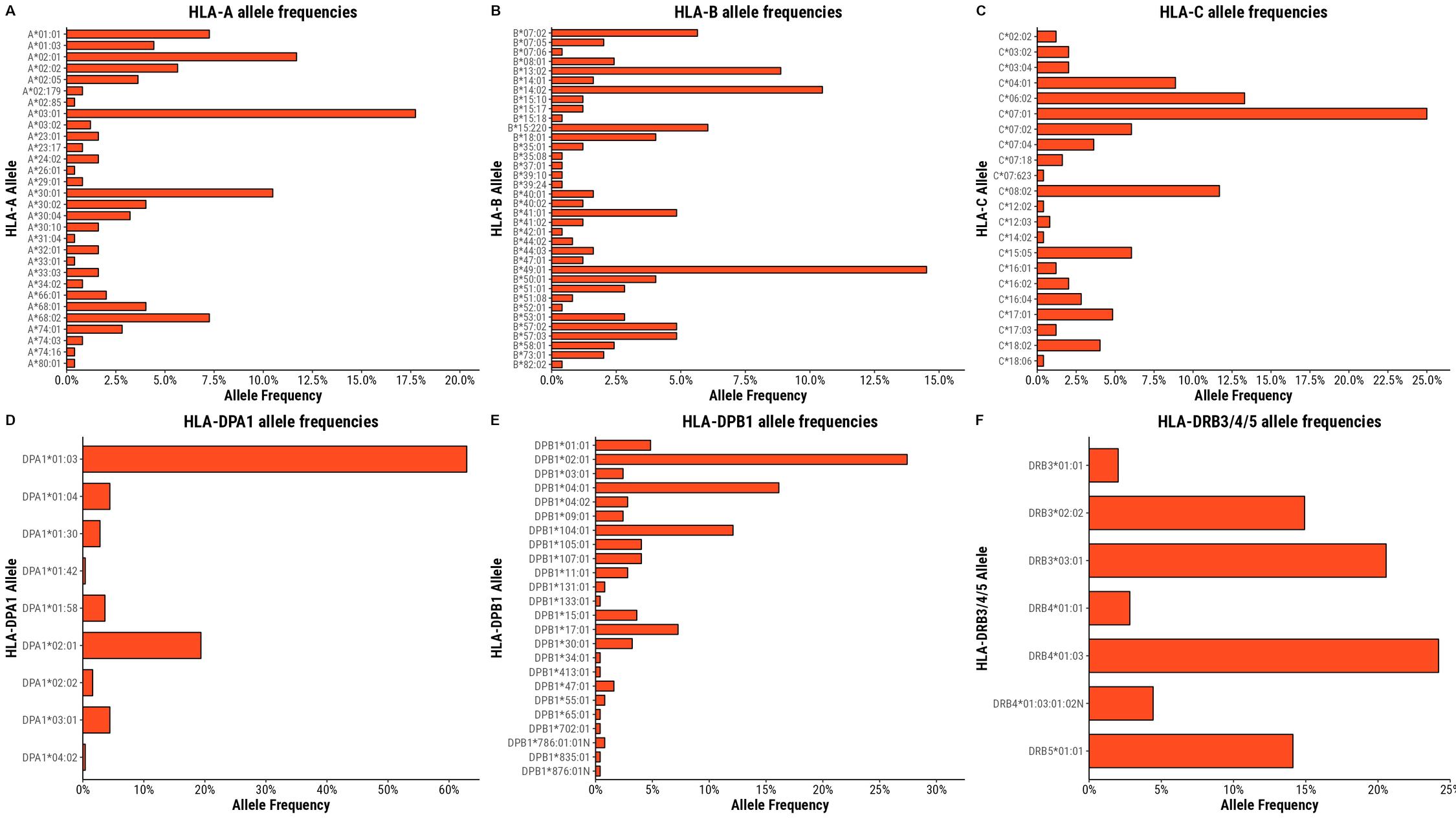

Supplement: S1 Fig — HLA allele frequencies in the study participants for A) HLA-A, B) HLA-B, C) HLA-C, D) HLA-DPA1, E) HLA-DPB1, and F) HLA-DRB3/4/5. The study participants (N = 124) were all adult HIV patients that were either positive for at least two Leishmania infection markers at any timepoint during the study (see methods), or that had a history of visceral leishmaniasis. We used the Oxford Nanopore Technologies sequencing-based NanoTYPE assay to detect all alleles spanning 11 different HLA genes. HLA-DRB1, HLA-DQA1, and HLA-DQB1 are listed in Fig 3 of the main manuscript. (TIF) [file pntd.0012000.s001.tif]

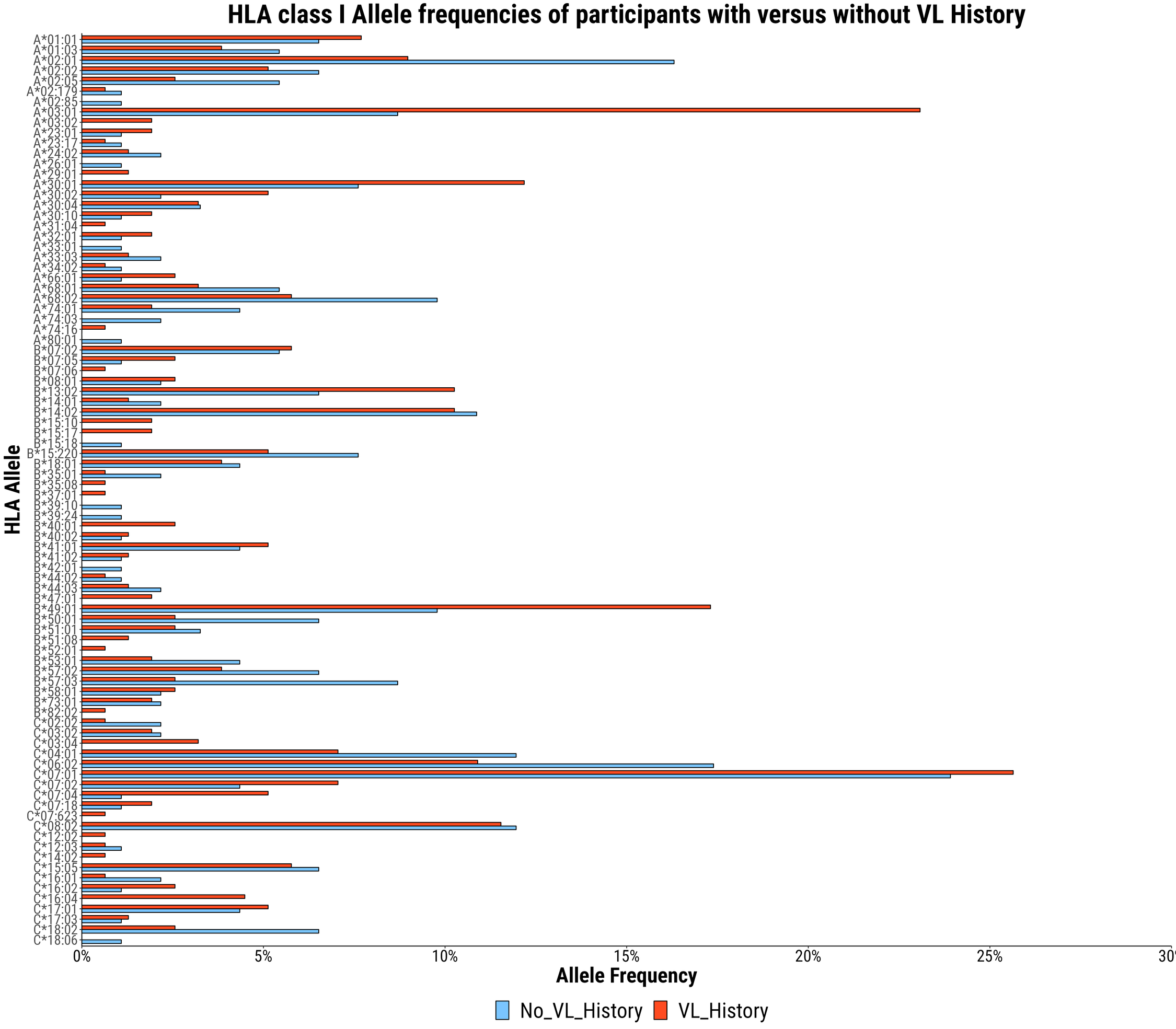

Supplement: S2 Fig — The study participants (N = 124) were all adult HIV patients that were either positive for at least two Leishmania infection markers at any timepoint during the study (No VL History, see methods), or that had a history of visceral leishmaniasis (VL history). We used the Oxford Nanopore Technologies sequencing-based NanoTYPE assay to detect all alleles spanning 11 different HLA genes. (TIF) [file pntd.0012000.s002.tif]

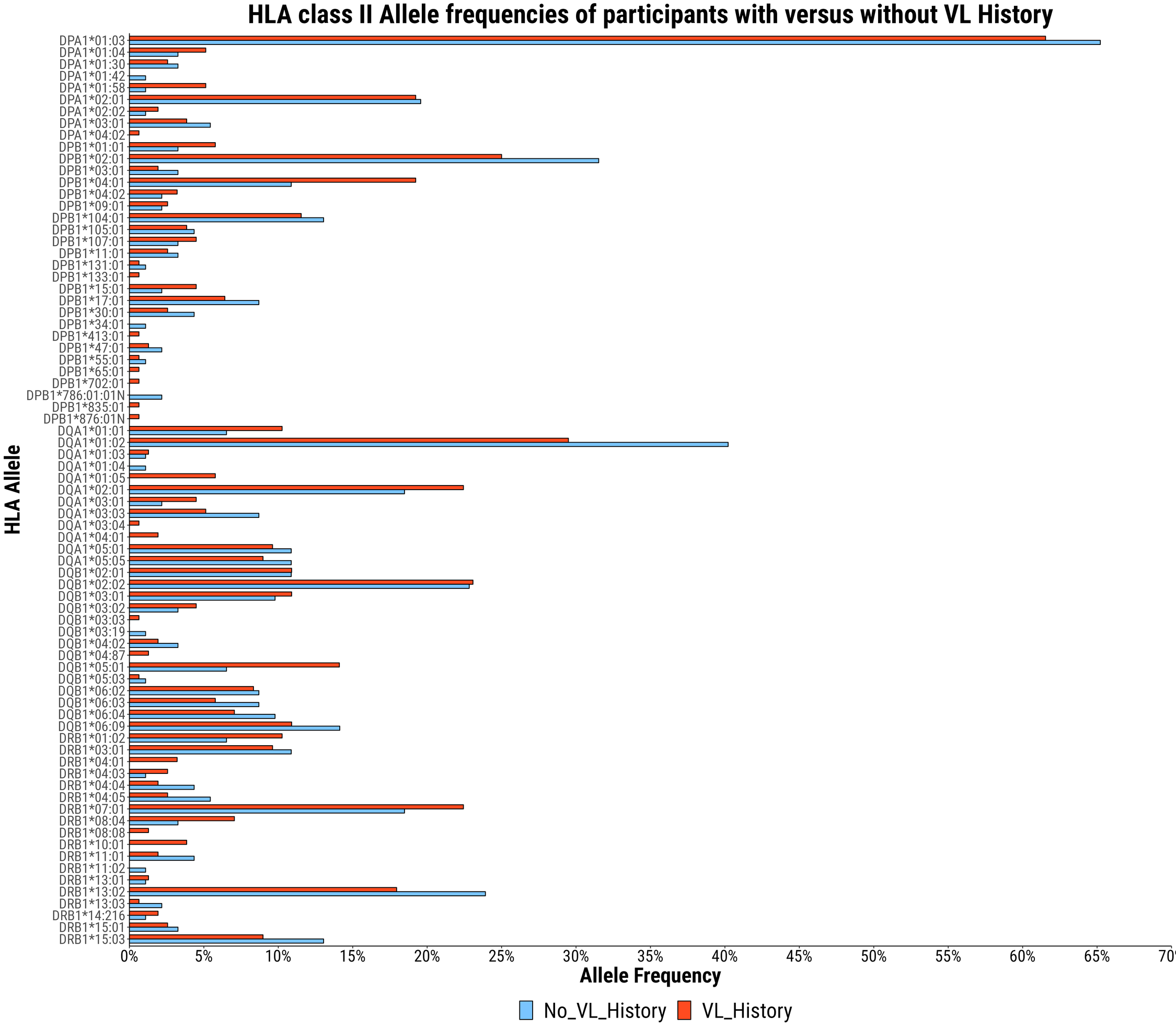

Supplement: S3 Fig — The study participants (N = 124) were all adult HIV patients that were either positive for at least two Leishmania infection markers at any timepoint during the study (No VL History, see methods), or that had a history of visceral leishmaniasis (VL history). We used the Oxford Nanopore Technologies sequencing-based NanoTYPE assay to detect all alleles spanning 11 different HLA genes. (TIF) [file pntd.0012000.s003.tif]
