# Supplementary material for: A preliminary indication that HLA-A*03:01 may be associated with visceral leishmaniasis development in people living with HIV in Ethiopia
Source: PLoS Negl Trop Dis. 2024 Sep 30;18(9):e0012000. doi: 10.1371/journal.pntd.0012000 (PMC11466428; doi:10.1371/journal.pntd.0012000)
Supplement: S1 Table — An OR above 1 indicates an increased risk for VL development, while an OR below 1 indicates increased protection against VL development. (DOCX) [file pntd.0012000.s004.docx]

**S1 Table |** All HLA alleles included in the HLA association analysis between past VL developers (n = 78) and asymptomatic Leishmania controllers (n = 46), ranked ascendingly by lowest p-value after Benjamini-Hochberg multiple testing correction. An OR above 1 indicates an increased risk for VL development, while an OR below 1 indicates increased protection against VL development.

| HLA allele | VL History (n,%) | No VL History (n,%) | OR (95%CI) | p-value | p_BH_-values |
| --- | --- | --- | --- | --- | --- |
| HLA-A*03:01 | 34 (43.6%) | 7 (15.2%) | 3.89 (1.62-9.36) | 0.0014 | 0.0170 |
| HLA-A*02:01 | 13 (16.7%) | 15 (32.6%) | 0.42 (0.18-0.98) | 0.0476 | 0.2856 |
| HLA-A*68:02 | 8 (10.3%) | 9 (19.6%) | 0.48 (0.18-1.29) | 0.1794 | 0.6775 |
| HLA-A*02:05 | 4 (5.1%) | 5 (10.9%) | 0.47 (0.13-1.62) | 0.2897 | 0.6775 |
| HLA-A*30:02 | 8 (10.3%) | 2 (4.3%) | 1.9 (0.49-7.4) | 0.3200 | 0.6775 |
| HLA-A*30:01 | 17 (21.8%) | 6 (13.0%) | 1.7 (0.65-4.43) | 0.3388 | 0.6775 |
| HLA-A*74:01 | 3 (3.8%) | 4 (8.7%) | 0.45 (0.12-1.78) | 0.4221 | 0.7011 |
| HLA-A*68:01 | 4 (5.1%) | 4 (8.7%) | 0.57 (0.16-2.09) | 0.4674 | 0.7011 |
| HLA-A*01:03 | 6 (7.7%) | 5 (10.9%) | 0.67 (0.21-2.13) | 0.5346 | 0.7128 |
| HLA-A*02:02 | 8 (10.3%) | 6 (13.0%) | 0.74 (0.26-2.14) | 0.7702 | 0.9242 |
| HLA-A*01:01 | 11 (14.1%) | 6 (13.0%) | 1.03 (0.38-2.84) | 1.0000 | 1.0000 |
| HLA-A*30:04 | 5 (6.4%) | 3 (6.5%) | 0.89 (0.24-3.34) | 1.0000 | 1.0000 |
| HLA-B*57:03 | 4 (5.1%) | 8 (17.4%) | 0.29 (0.09-0.92) | 0.0547 | 0.7654 |
| HLA-B*50:01 | 4 (5.1%) | 6 (13.0%) | 0.39 (0.12-1.31) | 0.1713 | 0.8442 |
| HLA-B*49:01 | 23 (29.5%) | 9 (19.6%) | 1.63 (0.7-3.79) | 0.2892 | 0.8442 |
| HLA-B*13:02 | 16 (20.5%) | 6 (13.0%) | 1.58 (0.6-4.14) | 0.3393 | 0.8442 |
| HLA-B*57:02 | 6 (7.7%) | 6 (13.0%) | 0.56 (0.18-1.71) | 0.3585 | 0.8442 |
| HLA-B*15:220 | 8 (10.3%) | 7 (15.2%) | 0.63 (0.23-1.77) | 0.4105 | 0.8442 |
| HLA-B*53:01 | 3 (3.8%) | 4 (8.7%) | 0.45 (0.12-1.78) | 0.4221 | 0.8442 |
| HLA-B*51:01 | 4 (5.1%) | 3 (6.5%) | 0.73 (0.19-2.88) | 0.7097 | 1.0000 |
| HLA-B*18:01 | 5 (6.4%) | 4 (8.7%) | 0.7 (0.2-2.42) | 0.7252 | 1.0000 |
| HLA-B*07:02 | 9 (11.5%) | 5 (10.9%) | 1.0 (0.34-2.95) | 1.0000 | 1.0000 |
| HLA-B*08:01 | 4 (5.1%) | 2 (4.3%) | 1.0 (0.23-4.38) | 1.0000 | 1.0000 |
| HLA-B*14:02 | 16 (20.5%) | 10 (21.7%) | 0.91 (0.38-2.14) | 1.0000 | 1.0000 |
| HLA-B*41:01 | 8 (10.3%) | 4 (8.7%) | 1.09 (0.34-3.47) | 1.0000 | 1.0000 |
| HLA-B*58:01 | 4 (5.1%) | 2 (4.3%) | 1.0 (0.23-4.38) | 1.0000 | 1.0000 |
| HLA-C*16:04 | 7 (9.0%) | 0 (0.0%) | 5.22 (0.63-43.12) | 0.0455 | 0.4551 |
| HLA-C*18:02 | 4 (5.1%) | 6 (13.0%) | 0.39 (0.12-1.31) | 0.1713 | 0.6304 |
| HLA-C*06:02 | 15 (19.2%) | 14 (30.4%) | 0.55 (0.24-1.25) | 0.1891 | 0.6304 |
| HLA-C*07:04 | 7 (9.0%) | 1 (2.2%) | 2.56 (0.52-12.57) | 0.2560 | 0.6400 |
| HLA-C*04:01 | 11 (14.1%) | 10 (21.7%) | 0.59 (0.24-1.48) | 0.3245 | 0.6490 |
| HLA-C*07:02 | 11 (14.1%) | 4 (8.7%) | 1.52 (0.5-4.61) | 0.5696 | 0.9494 |
| HLA-C*15:05 | 9 (11.5%) | 6 (13.0%) | 0.84 (0.3-2.37) | 0.7838 | 1.0000 |
| HLA-C*07:01 | 33 (42.3%) | 20 (43.5%) | 0.95 (0.46-1.96) | 1.0000 | 1.0000 |
| HLA-C*08:02 | 18 (23.1%) | 11 (23.9%) | 0.93 (0.41-2.15) | 1.0000 | 1.0000 |
| HLA-C*17:01 | 8 (10.3%) | 4 (8.7%) | 1.09 (0.34-3.47) | 1.0000 | 1.0000 |
| HLA-DQA1*01:05 | 9 (11.5%) | 0 (0.0%) | 6.71 (0.83-54.21) | 0.0257 | 0.2055 |
| HLA-DQA1*01:02 | 38 (48.7%) | 29 (63.0%) | 0.57 (0.28-1.18) | 0.1388 | 0.5550 |
| HLA-DQA1*01:01 | 16 (20.5%) | 5 (10.9%) | 1.89 (0.69-5.18) | 0.2180 | 0.5813 |
| HLA-DQA1*03:03 | 7 (9.0%) | 7 (15.2%) | 0.56 (0.19-1.59) | 0.3795 | 0.7591 |
| HLA-DQA1*03:01 | 7 (9.0%) | 2 (4.3%) | 1.67 (0.42-6.61) | 0.4824 | 0.7719 |
| HLA-DQA1*02:01 | 32 (41.0%) | 17 (37.0%) | 1.17 (0.56-2.44) | 0.7064 | 0.9419 |
| HLA-DQA1*05:01 | 15 (19.2%) | 9 (19.6%) | 0.95 (0.39-2.31) | 1.0000 | 1.0000 |
| HLA-DQA1*05:05 | 14 (17.9%) | 8 (17.4%) | 1.0 (0.4-2.5) | 1.0000 | 1.0000 |
| HLA-DQB1*05:01 | 21 (26.9%) | 5 (10.9%) | 2.66 (0.99-7.12) | 0.0405 | 0.4051 |
| HLA-DQB1*06:04 | 10 (12.8%) | 9 (19.6%) | 0.61 (0.24-1.56) | 0.3167 | 0.9641 |
| HLA-DQB1*06:09 | 16 (20.5%) | 13 (28.3%) | 0.66 (0.29-1.49) | 0.3818 | 0.9641 |
| HLA-DQB1*06:03 | 9 (11.5%) | 8 (17.4%) | 0.62 (0.23-1.65) | 0.4214 | 0.9641 |
| HLA-DQB1*03:01 | 17 (21.8%) | 7 (15.2%) | 1.45 (0.58-3.65) | 0.4820 | 0.9641 |
| HLA-DQB1*04:02 | 3 (3.8%) | 3 (6.5%) | 0.58 (0.14-2.43) | 0.6694 | 1.0000 |
| HLA-DQB1*03:02 | 7 (9.0%) | 3 (6.5%) | 1.22 (0.35-4.3) | 0.7431 | 1.0000 |
| HLA-DQB1*02:01 | 17 (21.8%) | 9 (19.6%) | 1.1 (0.46-2.64) | 0.8230 | 1.0000 |
| HLA-DQB1*02:02 | 32 (41.0%) | 19 (41.3%) | 0.98 (0.48-2.03) | 1.0000 | 1.0000 |
| HLA-DQB1*06:02 | 12 (15.4%) | 7 (15.2%) | 0.97 (0.37-2.54) | 1.0000 | 1.0000 |
| HLA-DPA1*01:58 | 8 (10.3%) | 1 (2.2%) | 2.92 (0.6-14.1) | 0.1519 | 0.9115 |
| HLA-DPA1*03:01 | 6 (7.7%) | 5 (10.9%) | 0.67 (0.21-2.13) | 0.5346 | 0.9189 |
| HLA-DPA1*01:30 | 4 (5.1%) | 3 (6.5%) | 0.73 (0.19-2.88) | 0.7097 | 0.9189 |
| HLA-DPA1*01:04 | 8 (10.3%) | 3 (6.5%) | 1.39 (0.4-4.8) | 0.7451 | 0.9189 |
| HLA-DPA1*01:03 | 69 (88.5%) | 42 (91.3%) | 0.81 (0.26-2.54) | 0.7657 | 0.9189 |
| HLA-DPA1*02:01 | 28 (35.9%) | 17 (37.0%) | 0.95 (0.45-1.99) | 1.0000 | 1.0000 |
| HLA-DPB1*02:01 | 33 (42.3%) | 26 (56.5%) | 0.57 (0.28-1.18) | 0.1401 | 0.9934 |
| HLA-DPB1*04:01 | 26 (33.3%) | 10 (21.7%) | 1.71 (0.76-3.88) | 0.2200 | 0.9934 |
| HLA-DPB1*30:01 | 4 (5.1%) | 4 (8.7%) | 0.57 (0.16-2.09) | 0.4674 | 0.9934 |
| HLA-DPB1*15:01 | 7 (9.0%) | 2 (4.3%) | 1.67 (0.42-6.61) | 0.4824 | 0.9934 |
| HLA-DPB1*17:01 | 10 (12.8%) | 8 (17.4%) | 0.69 (0.26-1.81) | 0.5989 | 0.9934 |
| HLA-DPB1*03:01 | 3 (3.8%) | 3 (6.5%) | 0.58 (0.14-2.43) | 0.6694 | 0.9934 |
| HLA-DPB1*11:01 | 3 (3.8%) | 3 (6.5%) | 0.58 (0.14-2.43) | 0.6694 | 0.9934 |
| HLA-DPB1*107:01 | 7 (9.0%) | 3 (6.5%) | 1.22 (0.35-4.3) | 0.7431 | 0.9934 |
| HLA-DPB1*01:01 | 8 (10.3%) | 3 (6.5%) | 1.39 (0.4-4.8) | 0.7451 | 0.9934 |
| HLA-DPB1*104:01 | 18 (23.1%) | 12 (26.1%) | 0.84 (0.37-1.9) | 0.8285 | 0.9942 |
| HLA-DPB1*04:02 | 5 (6.4%) | 2 (4.3%) | 1.22 (0.29-5.11) | 1.0000 | 1.0000 |
| HLA-DPB1*105:01 | 6 (7.7%) | 4 (8.7%) | 0.82 (0.25-2.76) | 1.0000 | 1.0000 |
| HLA-DRB1*10:01 | 6 (7.7%) | 0 (0.0%) | 4.51 (0.54-37.82) | 0.0836 | 0.6427 |
| HLA-DRB1*01:02 | 16 (20.5%) | 5 (10.9%) | 1.89 (0.69-5.18) | 0.2180 | 0.6427 |
| HLA-DRB1*13:02 | 24 (30.8%) | 19 (41.3%) | 0.64 (0.3-1.34) | 0.2477 | 0.6427 |
| HLA-DRB1*08:04 | 11 (14.1%) | 3 (6.5%) | 1.94 (0.59-6.4) | 0.2495 | 0.6427 |
| HLA-DRB1*15:03 | 13 (16.7%) | 11 (23.9%) | 0.64 (0.27-1.52) | 0.3530 | 0.6427 |
| HLA-DRB1*04:04 | 3 (3.8%) | 4 (8.7%) | 0.45 (0.12-1.78) | 0.4221 | 0.6427 |
| HLA-DRB1*11:01 | 3 (3.8%) | 4 (8.7%) | 0.45 (0.12-1.78) | 0.4221 | 0.6427 |
| HLA-DRB1*04:05 | 4 (5.1%) | 4 (8.7%) | 0.57 (0.16-2.09) | 0.4674 | 0.6427 |
| HLA-DRB1*07:01 | 32 (41.0%) | 17 (37.0%) | 1.17 (0.56-2.44) | 0.7064 | 0.7807 |
| HLA-DRB1*15:01 | 4 (5.1%) | 3 (6.5%) | 0.73 (0.19-2.88) | 0.7097 | 0.7807 |
| HLA-DRB1*03:01 | 15 (19.2%) | 9 (19.6%) | 0.95 (0.39-2.31) | 1.0000 | 1.0000 |
