# Supplementary material for: A preliminary indication that HLA-A*03:01 may be associated with visceral leishmaniasis development in people living with HIV in Ethiopia
Source: PLoS Negl Trop Dis. 2024 Sep 30;18(9):e0012000. doi: 10.1371/journal.pntd.0012000 (PMC11466428; doi:10.1371/journal.pntd.0012000)
Supplement: S2 Table — All alleles are reported up to the second field resolution (4-digit). Counts are the number of times an allele appears in the population. (DOCX) [file pntd.0012000.s005.docx]

**S2 Table |** The HLA allele frequencies of all HLA alleles detected in our study population of 124 Leishmania-infected and HIV co-infected individuals living in NW-Ethiopia. All alleles are reported up to the second field resolution (4-digit). Counts are the number of times an allele appears in the population.

| HLA allele | Counts | Allele Frequency |
| --- | --- | --- |
| A*01:01 | 18 | 0.072581 |
| A*01:03 | 11 | 0.044355 |
| A*02:01 | 29 | 0.116935 |
| A*02:02 | 14 | 0.056452 |
| A*02:05 | 9 | 0.03629 |
| A*02:179 | 2 | 0.008065 |
| A*02:85 | 1 | 0.004032 |
| A*03:01 | 44 | 0.177419 |
| A*03:02 | 3 | 0.012097 |
| A*23:01 | 4 | 0.016129 |
| A*23:17 | 2 | 0.008065 |
| A*24:02 | 4 | 0.016129 |
| A*26:01 | 1 | 0.004032 |
| A*29:01 | 2 | 0.008065 |
| A*30:01 | 26 | 0.104839 |
| A*30:02 | 10 | 0.040323 |
| A*30:04 | 8 | 0.032258 |
| A*30:10 | 4 | 0.016129 |
| A*31:04 | 1 | 0.004032 |
| A*32:01 | 4 | 0.016129 |
| A*33:01 | 1 | 0.004032 |
| A*33:03 | 4 | 0.016129 |
| A*34:02 | 2 | 0.008065 |
| A*66:01 | 5 | 0.020161 |
| A*68:01 | 10 | 0.040323 |
| A*68:02 | 18 | 0.072581 |
| A*74:01 | 7 | 0.028226 |
| A*74:03 | 2 | 0.008065 |
| A*74:16 | 1 | 0.004032 |
| A*80:01 | 1 | 0.004032 |
| B*07:02 | 14 | 0.056452 |
| B*07:05 | 5 | 0.020161 |
| B*07:06 | 1 | 0.004032 |
| B*08:01 | 6 | 0.024194 |
| B*13:02 | 22 | 0.08871 |
| B*14:01 | 4 | 0.016129 |
| B*14:02 | 26 | 0.104839 |
| B*15:10 | 3 | 0.012097 |
| B*15:17 | 3 | 0.012097 |
| B*15:18 | 1 | 0.004032 |
| B*15:220 | 15 | 0.060484 |
| B*18:01 | 10 | 0.040323 |
| B*35:01 | 3 | 0.012097 |
| B*35:08 | 1 | 0.004032 |
| B*37:01 | 1 | 0.004032 |
| B*39:10 | 1 | 0.004032 |
| B*39:24 | 1 | 0.004032 |
| B*40:01 | 4 | 0.016129 |
| B*40:02 | 3 | 0.012097 |
| B*41:01 | 12 | 0.048387 |
| B*41:02 | 3 | 0.012097 |
| B*42:01 | 1 | 0.004032 |
| B*44:02 | 2 | 0.008065 |
| B*44:03 | 4 | 0.016129 |
| B*47:01 | 3 | 0.012097 |
| B*49:01 | 36 | 0.145161 |
| B*50:01 | 10 | 0.040323 |
| B*51:01 | 7 | 0.028226 |
| B*51:08 | 2 | 0.008065 |
| B*52:01 | 1 | 0.004032 |
| B*53:01 | 7 | 0.028226 |
| B*57:02 | 12 | 0.048387 |
| B*57:03 | 12 | 0.048387 |
| B*58:01 | 6 | 0.024194 |
| B*73:01 | 5 | 0.020161 |
| B*82:02 | 1 | 0.004032 |
| C*02:02 | 3 | 0.012097 |
| C*03:02 | 5 | 0.020161 |
| C*03:04 | 5 | 0.020161 |
| C*04:01 | 22 | 0.08871 |
| C*06:02 | 33 | 0.133065 |
| C*07:01 | 62 | 0.25 |
| C*07:02 | 15 | 0.060484 |
| C*07:04 | 9 | 0.03629 |
| C*07:18 | 4 | 0.016129 |
| C*07:623 | 1 | 0.004032 |
| C*08:02 | 29 | 0.116935 |
| C*12:02 | 1 | 0.004032 |
| C*12:03 | 2 | 0.008065 |
| C*14:02 | 1 | 0.004032 |
| C*15:05 | 15 | 0.060484 |
| C*16:01 | 3 | 0.012097 |
| C*16:02 | 5 | 0.020161 |
| C*16:04 | 7 | 0.028226 |
| C*17:01 | 12 | 0.048387 |
| C*17:03 | 3 | 0.012097 |
| C*18:02 | 10 | 0.040323 |
| C*18:06 | 1 | 0.004032 |
| DPA1*01:03 | 156 | 0.629032 |
| DPA1*01:04 | 11 | 0.044355 |
| DPA1*01:30 | 7 | 0.028226 |
| DPA1*01:42 | 1 | 0.004032 |
| DPA1*01:58 | 9 | 0.03629 |
| DPA1*02:01 | 48 | 0.193548 |
| DPA1*02:02 | 4 | 0.016129 |
| DPA1*03:01 | 11 | 0.044355 |
| DPA1*04:02 | 1 | 0.004032 |
| DPB1*01:01 | 12 | 0.048387 |
| DPB1*02:01 | 68 | 0.274194 |
| DPB1*03:01 | 6 | 0.024194 |
| DPB1*04:01 | 40 | 0.16129 |
| DPB1*04:02 | 7 | 0.028226 |
| DPB1*09:01 | 6 | 0.024194 |
| DPB1*104:01 | 30 | 0.120968 |
| DPB1*105:01 | 10 | 0.040323 |
| DPB1*107:01 | 10 | 0.040323 |
| DPB1*11:01 | 7 | 0.028226 |
| DPB1*131:01 | 2 | 0.008065 |
| DPB1*133:01 | 1 | 0.004032 |
| DPB1*15:01 | 9 | 0.03629 |
| DPB1*17:01 | 18 | 0.072581 |
| DPB1*30:01 | 8 | 0.032258 |
| DPB1*34:01 | 1 | 0.004032 |
| DPB1*413:01 | 1 | 0.004032 |
| DPB1*47:01 | 4 | 0.016129 |
| DPB1*55:01 | 2 | 0.008065 |
| DPB1*65:01 | 1 | 0.004032 |
| DPB1*702:01 | 1 | 0.004032 |
| DPB1*786:01:01N | 2 | 0.008065 |
| DPB1*835:01 | 1 | 0.004032 |
| DPB1*876:01N | 1 | 0.004032 |
| DQA1*01:01 | 22 | 0.08871 |
| DQA1*01:02 | 83 | 0.334677 |
| DQA1*01:03 | 3 | 0.012097 |
| DQA1*01:04 | 1 | 0.004032 |
| DQA1*01:05 | 9 | 0.03629 |
| DQA1*02:01 | 52 | 0.209677 |
| DQA1*03:01 | 9 | 0.03629 |
| DQA1*03:03 | 16 | 0.064516 |
| DQA1*03:04 | 1 | 0.004032 |
| DQA1*04:01 | 3 | 0.012097 |
| DQA1*05:01 | 25 | 0.100806 |
| DQA1*05:05 | 24 | 0.096774 |
| DQB1*02:01 | 27 | 0.108871 |
| DQB1*02:02 | 57 | 0.229839 |
| DQB1*03:01 | 26 | 0.104839 |
| DQB1*03:02 | 10 | 0.040323 |
| DQB1*03:03 | 1 | 0.004032 |
| DQB1*03:19 | 1 | 0.004032 |
| DQB1*04:02 | 6 | 0.024194 |
| DQB1*04:87 | 2 | 0.008065 |
| DQB1*05:01 | 28 | 0.112903 |
| DQB1*05:03 | 2 | 0.008065 |
| DQB1*06:02 | 21 | 0.084677 |
| DQB1*06:03 | 17 | 0.068548 |
| DQB1*06:04 | 20 | 0.080645 |
| DQB1*06:09 | 30 | 0.120968 |
| DRB1*01:02 | 22 | 0.08871 |
| DRB1*03:01 | 25 | 0.100806 |
| DRB1*04:01 | 5 | 0.020161 |
| DRB1*04:03 | 5 | 0.020161 |
| DRB1*04:04 | 7 | 0.028226 |
| DRB1*04:05 | 9 | 0.03629 |
| DRB1*07:01 | 52 | 0.209677 |
| DRB1*08:04 | 14 | 0.056452 |
| DRB1*08:08 | 2 | 0.008065 |
| DRB1*10:01 | 6 | 0.024194 |
| DRB1*11:01 | 7 | 0.028226 |
| DRB1*11:02 | 1 | 0.004032 |
| DRB1*13:01 | 3 | 0.012097 |
| DRB1*13:02 | 50 | 0.201613 |
| DRB1*13:03 | 3 | 0.012097 |
| DRB1*14:216 | 4 | 0.016129 |
| DRB1*15:01 | 7 | 0.028226 |
| DRB1*15:03 | 26 | 0.104839 |
| DRB3*01:01 | 5 | 0.020161 |
| DRB3*02:02 | 37 | 0.149194 |
| DRB3*03:01 | 51 | 0.205645 |
| DRB4*01:01 | 7 | 0.028226 |
| DRB4*01:03 | 60 | 0.241935 |
| DRB4*01:03:01:02N | 11 | 0.044355 |
| DRB5*01:01 | 35 | 0.141129 |
