# Supplementary material for: A preliminary indication that HLA-A*03:01 may be associated with visceral leishmaniasis development in people living with HIV in Ethiopia
Source: PLoS Negl Trop Dis. 2024 Sep 30;18(9):e0012000. doi: 10.1371/journal.pntd.0012000 (PMC11466428; doi:10.1371/journal.pntd.0012000)
Supplement: Attachment — Submitted filename: PreLeisHLA_Letter_to_the_Reviewers.docx [file pntd.0012000.s006.docx]

Reviewer comments

Reviewer #1

Overall, the manuscript is well-written. Particularly intriguing is the comparison between ONT-based and short-read-based HLA typing, as the application of ONT-based HLA typing could be pivotal in Low- and Middle-Income Countries (LMICs).

Author response: We thank the reviewer for the recognition of our work.

1. Does the biobank consent exclusively cover consent for genetic testing? If feasible, please provide a copy of the consent form in the supplementary data for transparency and reference.

The informed consent did not *exclusively* cover consent for genetic testing, but did include genetic testing among other assays (see screenshot of ICF below).

The participants also consented to long-term storage of samples after completion of the main study, and that their samples could be used for future research to better understand how to diagnose and treat visceral leishmaniasis. The study protocol on biobanked samples along with the original ICFs were sent to the ethical boards listed in the manuscript for separate ethical approval. We are not inclined to add the original ICF as we believe this is covered by the ethical approval section. Alternatively, we have added an additional sentence on approval for testing of genetic material in the methods section of the manuscript.

2. Please ensure that all software used for data analysis is properly referenced.

We agree with the reviewer that all software should be properly referenced. It is, however, not typical to cite common programming languages nor to cite proprietary company software other than mentioning the company and country in-text. We have, however, added references to the R and Python programming languages, and have made sure that all other software is properly cited. We hope this appeases the reviewer.

3. Were the authors' study participants limited to a single ethnic group in Ethiopia, or were patients recruited from various ethnic backgrounds such as Oromos, Amharas, Somalis, etc.? Please provide further information in the manuscript.

Although the area often has young male migrants that work in crop cultivation, all participants included in this ancilliary study were permanent residents of the lowlands near the Abdurafi in the Amhara region (bordering Tigray) and were recruited at the Abdurafi Health Centre. Unfortunately, we did not gather other information about their ethnicity, and as such we cannot exclude that there were individuals of for example Tigray Oromo, or Somali descent, living in the Abdurafi region, that may have been included in the cohort. However, we do not believe it would be a large confounder as most participants are expected to be of Amhara descent and the HLA allele frequency distribution matched those of an earlier profiling study in the Amhara region (as mentioned in the results section). We have now further clarified this in the manuscript.

4. It's crucial to perform a Hardy-Weinberg test on control samples to assess potential population stratification.

We agree with the reviewer that a Hardy-Weinberg Equilibrium (HWE) test should have been performed. However, we argue that we do not have a true ‘control’ population, as we can expect that the asymptomatic *Leishmania* controller group may also have a biologically relevant HLA association that confers protection. Therefore, this is not a true ‘case-control’ study, as we expect both groups to be biologically relevant and associated with disease outcome. To be more stringent, we have performed a HWE on both groups, and did not see obvious population stratification. We have added this test to the methods and results section.

5. Did the authors conduct an HLA haplotype association test?

Prior to the initial submission, we had already performed an HLA haplotype association test. However, due to the high genetic diversity in a relatively low sample size cohort, all but one haplotypes were unique to individuals. As such, nothing would be significant in such a case. We have now included a sentence in the discussion to address this.

6. Considering weaker associated HLA alleles, have you considered using 2-digit alleles to increase the sample size in each comparison?

Prior to the initial submission, we had already performed the association analyses at the 2-digit level, however, results were similar to the 4-digit level. HLA-A*03 remained the only significantly associated allele. Thus, we chose to include only the higher resolution 4-digit level results, as this level defines most of the MHC epitope-binding repertoire. We have now added a sentence in the discussion section that the observed association persists on the 2-digit allele level.

7. In Figure 3, please provide separate bar-plots for allele frequencies of past VL and the asymptomatic cohort.

We agree with the reviewer that this is a valuable addition and thank the reviewer for their feedback. We have now added this in two separate figures in the supplementary material, and briefly mention these in-text in the results section.

# Reviewer #2

I strongly suggest to change the title, and hone down the asseverations of associations being made. This study provides some preliminary findings that may guide larger cohort studies that are properly powered to address the relevant question being asked.

Author response: We thank the reviewer for the detailed review and feedback. We agree that due to sample size limitations, our asseverations were too strong, and have been toned down accordingly throughout the manuscript (including the title). We appreciate the recognition that this study provides preliminary findings that could guide a larger cohort study.

1. It is not clear what the authors consider a VL case; VL patients with clinical manifestations, or symptomatic plus asymptomatic cases. This should be clearly stated because the epidemiological data they present (i.e. first paragraph of the introduction) can be misinterpreted.

We only consider VL patients with clinical manifestations as VL cases, and not asymptomatic individuals. This is why, in the first paragraph of the introduction, we only speak of “asymptomatic *Leishmania* infection” that can progress to “VL disease”. However, we have further clarified in-text that the presented epidemiological case data only concerns VL patients with clinical manifestations, and not asymptomatic individuals.

2. In an area where CL is also quite prevalent, how does “VL asymptomatic infections” are differentiated from pre-exposure to CL causing species in those that are rK39 negative?

We would like to clarify to the reviewer that while CL is prevalent in Ethiopia, this is typically not in the same regions as where VL is prevalent. The vector for VL-causing species particularly occurs in the lowlands, while the vector for the CL-causing species occurs in the highlands. However, most participants were stable/permanent residents of the lowland areas and only few migrants (more likely to have been exposed to CL) were included in the study. However, even then, we can not exclude that participants may have been pre-exposed to CL while remaining in other areas, and we have not differentiated these during our study. It should be mentioned, though, that no participant developed active cutaneous lesions during the long-term follow-up, although we did not verify CL scarring. As such, while we do not expect it to be a major limitation, we have added a small sentence on this in the discussion.

3. In the intro the authors comment: “The asymptomatic stage preceding VL disease is detectable by a variety of Leishmania infection markers, and can thus provide an opportune moment to screen for those at risk for VL development and to initiate preventative strategies (4, 7, 8)”. Please define which are these methods and their relevance in discriminating active infection, immunological memory of infection, and pre-exposure to different Leishmania species.

We believe the diversity of assays to measure a *Leishmania* infection and their role in discrimination of disease stages and/or outcomes is a separate discussion field. Moreover, that is the topic of the main clinical manuscript of this PreLeisH cohort (currently under review at eBioMedicine). As this manuscript was focused on the HLA associations, we hope the reviewer can agree with us only referencing to relevant papers on this topic. We can, however, briefly reflect here on the *Leishmania* infection markers used in this manuscript. In East Africa (also in HIV-positive individuals), the rK39 ELISA, rK39 RDT, DAT, kaTEX, and PCR are often used for VL diagnosis. While the serological methods (rK39 ELISA, rK39 RDT, and DAT) are quite sensitive and specific for VL case diagnosis (less so in East-Africa than in India https://doi.org/10.4269%2Fajtmh.13-0759), the antibodies are detectable for a longer period of time after infection. In addition, exposure to different *Leishmania* species may also elicit antibodies. Thus, the serological methods are less relevant for identifying active VL (relapse or re-infection) cases in those that have a prior history of VL. In contrast, kaTEX, a *Leishmania* antigen urine test, together with PCR-based methods, are more direct markers of active infection (this was observed in the main PreLeisH manuscript) and VL disease development. In any case, as was already initially mentioned in the manuscript discussion, we opted for positivity to at least two *Leishmania* infection markers as inclusion criteria for the asymptomatic group, to be more stringent.

4. The selection of study groups may introduce noise to the real association of HLA variants and disease progression. Although the team is contrasting patients who had experienced VL, vs. those that are asymptomatic, it is unknown whether the VL group was asymptomatic prior to developing disease. No prospective follow-up of this cohort was done prior to onset of symptoms, which begs the question of their infection status prior to developing VL. To really identify markers associated with disease progression, the prospective cohort should start from all asymptomatic patients, with rigorous follow-up to identify onset of symptoms in a given time period. That would clearly define the study groups.

We agree with the reviewer that there is some inherent noise in any such comparison (albeit we argue less than comparing versus healthy endemic controls). While we can assume most VL patients were asymptomatic at one point preceding disease, it is indeed unknown how long the VL group was asymptomatic prior to developing disease. Thus, we cannot be sure that some of the asymptomatic *Leishmania* ‘controllers’ may not end up developing VL in the future. However, we would like to clarify that the PreLeisH main study was a prospective cohort study such as proposed by the reviewer, starting out with HIV+ participants without any current VL disease, and that the asymptomatic *Leishmania*-infected individuals were followed-up for a median of 22 months. These participants did not develop any symptoms in these years, and some even reverted from positive infection markers to negativity. We know from prior work that around 40% of *Leishmania* infection marker-positive individuals end up reverting to negative markers within 12 months, and that only a very small fraction of asymptomatic *Leishmania*-infected individuals actually develop VL symptoms (https://doi.org/10.1371%2Fjournal.pntd.0007765). Given these facts, we expect that it would only be a very minor part of the asymptomatic *Leishmania* controller group that may end up developing VL somewhere in the future after our already long follow-up. However, we acknowledge that this is still a limitation, and have now addressed this in the discussion. In addition, in line with comment #1, we have toned down our asseverations based on the mentioned limitations, as we fully agree these are preliminary findings that need validation in larger cohort studies.

5. It is unclear how the sample size is suitable for the proposed analyses. A total of 83 alleles were analyzed in a sample size of 124 patients. The likelihood for spurious associations is very high. The authors mention in the methods that a previous study with some HLA frequencies in Ethiopian populations. Why not use this at least as a starting point for sample size estimation? Please include proper sample size estimations and the statistical support for the effect given the sample size and multiple testing.

We agree that the sample size is relatively low due to the restricted number of biobanked samples and cohort limitations, and have accordingly adapted our title and toned down our conclusions. However, in general, the sample size estimations/power calculations as proposed by the reviewer are often rather used to calculate power to detect differences, and do not control for spurious associations. This is what multiple testing is used for, which we performed using a false-discovery rate controlling method (Benjamini-Hochberg), which was already mentioned in the initially submitted manuscript. Nevertheless, we conducted a post hoc power calculation which showed we had at least 80% power to find associations for which the true Odd’s Ratio is 3.3 or more. Lastly, the previously published study to which the reviewer refers to did not perform any association analyses, and performs HLA genotyping only targeted to certain loci and alleles, making it unsuitable for our sample size estimations. It is of note, that we included more individuals (n=124) compared to this previous study (n=98).

6. Are there effects associated with possible admixture of the populations? No description of the ethnic background was included for this cohort. Please include in the demographics table description of ethnicity/race.

We thank the reviewer for raising a topic that was not properly addressed yet in the manuscript. All participants of this ancilliary study were permanent/stable residents of the lowlands near the Abdurafi region of Amhara province (bordering Tigray) and were recruited at the Abdurafi Health Centre. Unfortunately, we did not gather other information about their ethnicity, and as such we cannot exclude that there were individuals of for example Tigrayan, Oromo, or Somali descent, living in the Abdurafi region, that may have been included in the cohort. However, we do not believe it would be a large confounder as the HLA allele frequency distribution matched those of an earlier profiling study in the Amhara region (as mentioned in the results section). We have now further clarified this in the manuscript.

7. There is over-interpretation od data, for example in the results section “Taken together, the strong association between HLA-A*03:01 and VL development, and the high prevalence of HLA-A*03:01 in the past VL group, suggests prognostic value in capturing those high at risk for VL in a clinical algorithm”. How can this be ascertained if no proper sample size estimations, control for confounding variables such as genetic background, among others, were made?

We thank the reviewer for this comment, and have toned down our wording throughout the manuscript, including the title. We believe we have sufficiently addressed the concerns according to sample size estimations and genetic background in the other comments.

8. Protocol approval numbers should be included

We thank the reviewer for their comment, and have now included protocol/IRB approval numbers.

9. Datasets could be made available in a de-identified manner, including HLA genotypes and at least group classifications

We agree with the reviewer that datasets could and should be made available. We planned to do so upon revisions, and have now made the datasets available on Zenodo (https://doi.org/10.5281/zenodo.12516076). However, we will not publish the raw sequencing data as this was a condition for ITM IRB ethical approval.

10. Is there any association between the HLA-A*03:01 and the outcome of HIV infection? How does having HIV affect the development of VL according to HLA? Is this same difference expected to occur in a non-HIV populations. All these aspects need to be discussed in the paper.

We agree with the reviewer that these are relevant questions, however, we could not identify relevant data on these aspects. Within our study, we were restricted to VL investigations as most participants were under antiretroviral therapy and had their HIV infection suppressed. In addition, for the past VL group we did not have their HIV-associated parameters (such as CD4 Count or HIV viral load) at time of VL development (prior to the study). However, a study in Ghana identified HLA-A*03:01-associated HIV mutations in the *gag* and *viv* genes (https://doi.org/10.7883/yoken.jjid.2019.201) that could potentially impact the host T cell response to HIV, but any such link is purely speculative and may not hold under antiretroviral therapy. Thus, we believe we can not sufficiently answer these questions. We have added this in as a limitation in the discussion.
