# Supplementary material for: A preliminary indication that HLA-A*03:01 may be associated with visceral leishmaniasis development in people living with HIV in Ethiopia
Source: PLoS Negl Trop Dis. 2024 Sep 30;18(9):e0012000. doi: 10.1371/journal.pntd.0012000 (PMC11466428; doi:10.1371/journal.pntd.0012000)
Supplement: Attachment — Submitted filename: PreLeisHLA_Letter_to_the_Reviewers.docx [file pntd.0012000.s007.docx]

Reviewer comments

Reviewer #3

Overall, the manuscript is well-qualified and has novelty. However, several flaws must adequately be taken care of.

Author response: We thank the reviewer for the recognition of our work.

1. In the M&M, Fig 1 should be explained in detail. Fig 1 should be edited graphically and have harmony in their color classification. For example, choose a specific color for No sample available, and so on. Don’t use italics in the flowchart. They should edit the fellow chart entirely based on the same literature in the PLOS NTD.

We agree with the reviewer that Figure 1 should be explained more in detail, and could benefit from graphical editing. We have adapted the figure to include most of the reviewer’s suggestion, but we want to clarify that PLOS NTD does not have a house-style nor policy for flow charts, and flow charts differ among publications. As such we have clarified the figure more in the legend, have removed the italics, now use dashed boxes for exclusions, have harmonized the color scheme, and have removed the name ‘asymptomatics’ for the n=126 group (as mentioned in the feedback below).

2. The inclusion criteria and exclusion criteria should be defined in the text.

The inclusion criteria and exclusion criteria of the main study have already been described on ClinicalTrials.gov as mentioned in the manuscript (Accession provided in the text). Nonetheless, we have added the following sentence describing the inclusion/exclusion criteria in the already existing ‘Study population and design’’ section:

“The inclusion criteria for the main PreLeisH study were a confirmation of HIV-positivity, and being enrolled in this active HIV treatment program. Exclusion criteria were being under 18 years old, having an active VL diagnosis at time of enrolment, inability to provide informed consent, and a medical emergency or other chronic medical conditions that made adherence to or participation in the study unlikely or inadvisable.”

In addition, we further clarified the selection of asymptomatic individuals (see comments below).

3. How did they recognize asymptomatic cases?

Due to the lack of a standardized way to define asymptomatic cases, we applied a strict definition as mentioned in the ‘Study population and design’ section. We defined the asymptomatic individuals as those having at least two *Leishmania* infection markers positive at any timepoint during the study (markers included rK39 RDT, rK39 ELISA, DAT, a *Leishmania* antigenuria-based latex agglutination test (KAtex), or a *Leishmania* PCR), and who remained asymptomatic during the complete study period. In other words, individuals that were clearly infected but did not have nor develop any symptoms for the complete study period. We also discussed in the discussion section that we opted for this stringent criterium of positivity on at least two *Leishmania* infection marker due to the lack of a golden standard test for asymptomatic *Leishmania* infection (and due to the risk of false positives). However, as this may have been unclear, we have further clarified this in the ‘Study population and design’ section.

4. The asymptomatic N: 126 was not included; explain it in the manuscript.

These individuals were excluded because they did not test positive for at least two *Leishmania* infection markers, as defined above and in the ‘Study population and design’ section. These individuals tested positive for only one *Leishmania* marker, which we believe does not constitute a robust ‘asymptomatic’ individual. Therefore, we now further clarified this in text, and have removed the naming ‘asymptomatic’ for this group from the figure.

5. Please mention the statistical analysis in detail. How did they evaluate the normality of the quantitative variables in each group?

The statistical analyses were mentioned in detail in the section “Statistics and HLA association analysis”. Because most variables were categorical and binary, which are by definition not normally distributed, the Fisher’s Exact test was performed. The only two exceptions are the quantitative variables ‘Age’ (skewed by inclusion criteria of 18 years or older) and ‘BMI’. However, due to the nature of Age and BMI as variables, they are typically not normally distributed in the first place and we did not test for normality initially. However, per the reviewer’s suggestion we have now evaluated the normality of Age and BMI using the Shapiro-Wilk test, resulting in *p*-values of 0.00028 and 0.001854 for Age and BMI, respectively. Thus, these variables are not normally distributed as expected. This has been included in the section “Statistics and HLA association analysis” section.

6. As they used PCR, they should mention the etiological agent of VL. I can’t find the species of VL in the results.

No species typing was performed in our study, only a PCR for presence of *Leishmania* kinetoplast DNA. However, the main etiological agent of VL in Ethiopia is known to be *L. donovani*. In accordance, we have added a sentence in the methods to describe the PCR in more detail, and added the species restriction as a limitation in the discussion.

7. Did they evaluate viscerotropic CL that can be detected in HIV-positive patients?

We thank the reviewer for this comment. As mentioned in the discussion as a limitation, we unfortunately did not assess prior exposure of participants to CL-causing *Leishmania* species nor to CL scars, although no participants developed CL lesions during the course of our study. Thus, while we did not evaluate viscerotropic CL in particular, we do not expect the participants to have been widely exposed to CL. This is due to vector dynamics: While CL (caused by L.aethiopica) is prevalent in Ethiopia, this is typically not in the same regions as where VL is prevalent. The vector for VL-causing species particularly is restricted to the lowlands, while the vector for the CL-causing species is restricted to the highlands. In addition, most participants were stable/permanent residents of the lowland areas and only few migrants from the highlands (more likely to have been exposed to CL) were included in the study.

8. Please mention the Leishmaniasis-associated HLA alleles derived from the literature in the discussion. Were there any differences between VL caused by L. infantum or L. donovani and also CL, MCL, DCL, or VL caused by L. tropica? Did the author find the alleles in common, especially in viscerotropic infections or severity of infection, symptomatic and asymptotic forms? Please explain them in the discussion.

As the manuscript was focused on VL, relevant VL-associated HLA alleles derived from the literature were already discussed in the results (See table 4) and discussion sections. With regard to CL, there are no reports of HLA associations with *L. tropica* (<https://doi.org/10.3390/vaccines9030270>) as of yet, so this can not be included. Similarly, no reports on HLA associations for viscerotropic infections have been published as of yet, and there are no HLA alleles associated with severity of disease for any form of leishmaniasis. To broaden our discussion to CL-associated HLA alleles, we have added a small sentence in the discussion to compare our observed HLA associations with the HLA alleles known to be associated with CL development:
“None of the observed significantly different HLA alleles in this study on VL matched the previously described cutaneous leishmaniasis-associated HLA alleles (15). However, these studies were mostly conducted in South-America which may not share HLA allele occurrence, with the exception of a study in Egypt, and were all conducted on CL-causing species, which may not share common antigens with VL-causing species.”
